# ASIAN AMERICAN FAMILY CAREGIVING EXPERIENCES COMPARED TO CAREGIVERS OF OTHER RACIAL/ETHNIC GROUPS

**DOI:** 10.1093/geroni/igac059.2355

**Published:** 2022-12-20

**Authors:** Christina Miyawaki, Erin Bouldin

**Affiliations:** University of Houston, Houston, Texas, United States; University of Utah, Salt Lake City, Utah, United States

## Abstract

Asian Americans (AAs) are the fastest-growing immigrant group in the U.S. While otherwise highly heterogeneous, AAs value filial piety and eldercare. This study compared the prevalence, health, and caregiving experiences of AA caregivers (18+ years) to AA non-caregivers and caregivers in other racial/ethnic groups. Data from the 2015-2019 Behavioral Risk Factor Surveillance System were used (N=252,602). Caregivers were classified as those who provided care/assistance to a parent/parent-in-law/grandparent with a long-term illness/disability. Chi-square tests were used to compare weighted estimates. Among 4,564 AAs, 4.1% (n=321) were caregivers. They were single (55.6%), college-educated (84.2%), working (64.7%), young (< 45 years)(68.2%), male (60.0%), and in excellent/very good/good health (86.3%). Compared to non-caregivers, caregivers were more often unmarried with more chronic health conditions. They assisted a parent/parent-in-law (73.4%) with personal care (52.7%) and household tasks (77.9%). Although overall caregiving experiences were similar across racial/ethnic groups, the prevalence of caregivers was higher among other racial/ethnic groups than AAs (8.3%, Hispanic to 14.8%, American Indian/Alaska Native, p< 0.001), which was a surprise due to their caregiving culture. Because caregiving is expected, they may not identify themselves as caregivers. The data were collected in English and Spanish but 44% of AAs had limited English proficiency. Those limitations may have affected the sample size. Despite the limitations, this study was unique because of the comparison among different racial/ethnic groups of caregivers across the nation. However, to examine a full picture of AA caregivers, we need to administer surveys for each AA ethnic group in various languages.

